# Touch of Chemokines

**DOI:** 10.3389/fimmu.2012.00175

**Published:** 2012-07-12

**Authors:** Xavier Blanchet, Marcella Langer, Christian Weber, Rory R. Koenen, Philipp von Hundelshausen

**Affiliations:** ^1^Institute for Cardiovascular Prevention, Ludwig-Maximilians-University of MunichMunich, Germany; ^2^Cardiovascular Research Institute Maastricht, Maastricht UniversityMaastricht, Netherlands; ^3^Munich Heart AllianceMunich, Germany

**Keywords:** chemokine, chemokine receptor, arrest, oligomerization, vascular inflammatory diseases, leukocyte trafficking, therapeutics

## Abstract

Chemoattractant cytokines or chemokines constitute a family of structurally related proteins found in vertebrates, bacteria, or viruses. So far, 48 chemokine genes have been identified in humans, which bind to around 20 chemokine receptors. These receptors belong to the seven transmembrane G-protein-coupled receptor family. Chemokines and their receptors were originally studied for their role in cellular trafficking of leukocytes during inflammation and immune surveillance. It is now known that they exert different functions under physiological conditions such as homeostasis, development, tissue repair, and angiogenesis but also under pathological disorders including tumorigenesis, cancer metastasis, inflammatory, and autoimmune diseases. Physicochemical properties of chemokines and chemokine receptors confer the ability to homo- and hetero-oligomerize. Many efforts are currently performed in establishing new therapeutically compounds able to target the chemokine/chemokine receptor system. In this review, we are interested in the role of chemokines in inflammatory disease and leukocyte trafficking with a focus on vascular inflammatory diseases, the operating synergism, and the emerging therapeutic approaches of chemokines.

## Introduction

With the exceptions of CX3CL1/fractalkine and CXCL16/SR-PSOX, chemoattractant cytokines or chemokines constitute a family of small soluble signaling molecules of approximately 70 amino acid residues with a molecular weight of 7–12 kDa. In addition to their monomeric form, these proteins are able to associate, forming dimers, tetramers, or multimers (i.e., to oligomerize). Chemokines have crucial roles in both homeostasis and disease. Their homeostatic roles include leukocyte maturation and trafficking, development, tissue repair, and angiogenesis (Ransohoff, 2009). As disease modulators, chemokines have roles in a wide variety of inflammatory and immune responses through the chemoattraction of innate and adaptive immune cells. To date, around 50 chemokines have been identified in humans, which have been grouped into one of four families, CXC, CC, CX3C, and XC, based on the arrangement of cysteine residues involved in the formation of disulfide bonds (Table 1). In the CXC and CX3C chemokine family, one or three amino acid residues are inserted between the first two of four cysteine residues, respectively. The first and third cysteine residues are absent in the XC subfamily that possesses only one disulfide bond. In the CC subfamily, the first two cysteines are juxtaposed. Another family has been recently described in the zebrafish genome, namely the CX family, which lacks one of the four cysteine residues highly conserved amongst chemokines (Nomiyama et al., 2008). All chemokines arose from a single ancestral gene, originating approximately 650 million years ago (Nomiyama et al., 2010). Amongst vertebrates, the zebrafish genome has the highest number of chemokine genes with more than 100 genes while both pufferfish Tetraodon and Fugu genomes contain less than 20 chemokine genes each. The human genome encompasses more than 50 different chemokine genes and pseudo-genes. These genes have undergone a rapid evolution in both their sequences and their family gene size. The conventional name is still often used, which may lead to some confusion while the International Union of Immunological Societies/World Health Organization Subcommittee on Chemokine Nomenclature has assigned a name to each chemokine and chemokine receptor (Bacon et al., 2001). A large number of human chemokine genes are known to be clustered on specific chromosomal regions. There are two major gene clusters comprising exclusively either CXC or CC genes on chromosome 4q13.3-q21.1 and 17q12, respectively (Table 1). These major clusters can be subdivided into two regions. For the CXC gene cluster, the regions are named GRO and IP10 while the regions of the CC gene cluster are called MCP and MIP (Nomiyama et al., 2010). The GRO region contains the CXCL1–CXCL8 genes and the IP10 region the CXCL9–CXCL13 genes, respectively. In the CC major cluster, the MCP and MIP regions comprise 6 and 12 genes, respectively (CCL2, CCL7, CCL11, CCL8, CCL13, CCL1 versus CCL5, CCL16, CCL14, CCL15, CCL23, CCL18, CCL3, CCL4, CCL3L3, CCL4L1, CCL3L1, CCL4L2). In addition to the two major clusters, a CC “mini”-cluster is found on chromosome 7 (comprising the CCL26 and CCL24 genes), on chromosome 9 (CCL27, CCL19, CCL21), and on chromosome 16 (CCL22, CX3CL1, and CCL17), respectively. Both XCL1 and XCL2 are also found in a “mini”-cluster on chromosome 1.

**Table 1:** **Human chemokine genes**.

Name	Official symbol	Conventional name	Chromosome	Gene size (kb)	Number of exons	Number of amino acids (mature form)	Cluster name	Receptor^1^
**CCL**								
CCL1	CCL1	TCA3; I-309	17q11.2	2.85	3	73	MCP	CCR8
CCL2	CCL2	MCP-1; MCAF; JE	17q11.2-q21.1	1.93	3	76	MCP	CCR2, CCR3, DARC, CCBP2
CCL3	CCL3	MIP-1α; LD78α	17q12	1.90	3	69	MIP	CCR1, CCR5, CCBP2
CCL3L1	CCL3L1	LD78β	17q12	1.89	3	70	MIP	CCR1, CCR5
CCL3P1	CCL3L2	–	17q21.1	–			MIP	
CCL3L3	CCL3L3	LD78β	17q12	1.88	3	70	MIP	CCR1, CCR5
CCL4	CCL4	MIP-1β	17q21-q23	1.79	3	69	MIP	CCR5, CCBP2
CCL4L1	CCL4L1	LAG-1	17q12	1.80	3	69	MIP	CCR5, CCBP2
CCL4L2	CCL4L2	LAG-1	17q12	1.80	3	68	MIP	CCR5, CCBP2
CCL5	CCL5	RANTES	17q11.2-q12	8.88	3	68	MIP	CCR1, CCR3, CCR5, DARC, CCBP2, CCRL2
CCL7	CCL7	MCP-3; MARC	17q11.2-q12	2.01	3	76	MCP	CCR1, CCR2, CCR3, CCR5, DARC, CCBP2
CCL8	CCL8	MCP-2	17q11.2	2.35	3	76	MCP	CCR2,CCR3
CCL11	CCL11	Eotaxin,	17q21.1-q21.2	2.51	3	74	MCP	CCR3,CCR5, DARC, D6
CCL13	CCL13	MCP-4	17q11.2	2.21	3	82	MCP	CCR2, CCR3, CCR5, DARC, CCBP2
CCL14	CCL14	HCC-1	17q11.2	3.07	4	74	MIP	CCR1
CCL15	CCL15	HCC-2	17q11.2	4.46	4	92	MIP	CCR1, CCR3, DARC
CCL16	CCL16	HCC-4; LEC	17q11.2	4.98	3	97	MIP	CCR1
CCL17	CCL17	TARC; ABCD-2	16q13	11.29	3	71	“Mini”-CC 16	CCR4, DARC, CCBP2
CCL18	CCL18	DC-CK1; PARC; AMAC-1	17q11.2	7.2	3	69	MIP	DARC
CCL19	CCL19	MIP-3β; ELC; Exodus-3	9p13	1.71	4	77	“Mini”-CC 9	CCR7, CCRL1, CCRL2
CCL20	CCL20	MIP-3α; LARC; Exodus-1	2q33-q37	3.72	4	70, 69		CCR6
CCL21	CCL21	6Ckine; SLC; Exodus-2	9p13	1.14	4	111	“Mini”-CC 9	CCR7,CCRL1
CCL22	CCL22	MDC; STCP-1; AMCD-1	16q13	7.41	3	69	“Mini”-CC 16	CCR4, DARC, CCBP2
CCL23	CCL23	CKβ8; MPIF-1	17q11.2	4.91	4	99, 116	MIP	CCR1, FPR2
CCL24	CCL24	Eotaxin-2; MPIF-2	7q11.23	1.92	3	93	“Mini”-CC 7	CCR3
CCL25	CCL25	TECK	19p13.2	34.81	5	127, 61		CCR9, CCRL1
CCL26	CCL26	Eotaxin-3, MIP-4α, IMAC	7q11.2	20.22	3	71	“Mini”-CC 7	CCR3
CCL27	CCL27	CTACK; ILC; ESKINE	9p13	0.80	3	88	“Mini”-CC 9	CCR10
CCL28	CCL28	MEC	5p12	30.88	3	108		CCR3, CCR10
**CXC**								
CXCL1	CXCL1	GRO-α; MGSA-α; MIP-2; KC	4q13.3	1.85	4	73	GRO	CXCR2, DARC
p-CXCL1	CXCL1P	–	4q13.3	–	4		GRO	
CXCL2	CXCL2	GRO-β; MGSA-β; MIP-2α	4q13.3	2.24	4	73	GRO	CXCR2, DARC
CXCL3	CXCL3	GRO-γ, MGSA-γ; MIP-2β	4q13.3	2.18	3	73	GRO	CXCR2, DARC
CXCL4	PF4	PF4	4q13.3	0.92	3	70	GRO	CXCR3
CXCL4L1	PF4V1	PF4-ALT; CXCL4V1	4q13.3	1.18	4	70	GRO	
CXCL5	CXCL5	ENA-78	4q13.3	3.05	4	78	GRO	CXCR2, DARC
CXCL6	CXCL6	GCP-2	4q13.3	2.20	3	77	GRO	CXCR1, CXCR2, DARC
CXCL7	PPBP	NAP-2; beta-TG; CTAP-III	4q13.3	1.75	3	81, 85, 94	GRO	CXCR2, DARC
p-CXCL7	PPBPL1	–	4q13.3	–			GRO	
CXCL8	IL8	IL8	4q13.3	3.21	4	79, 82	GRO	CXCR1, CXCR2, DARC
CXCL9	CXCL9	MIG	4q21.1	6.02	4	103	IP10	CXCR3
CXCL10	CXCL10	IP10; CRG-2	4q21.1	2.42	4	77	IP10	CXCR3
CXCL11	CXCL11	I-TAC,	4q21.1	2.51	4	73	IP10	CXCR3, CXCR7, DARC
CXCL12	CXCL12	SDF-1α	10q11.1	14.94	4	68		CXCR4, CXCR7
CXCL12	CXCL12	SDF-1β	–	–	–	72		
CXCL12	CXCL12	SDF-1γ	–	–	–	98		
CXCL13	CXCL13	BCA-1; BLC	4q21.1	100	4	87	IP10	CXCR5
CXCL14	CXCL14	BRAK	5q31	8.60	4	77		?
CXCL16	CXCL16	SR-PSOX	17p13.2	6.39	5	225^2^		CXCR6
CXCL17	CXCL17	DMC	19q13.2	14.44	4	98		?
**XC**								
XCL1	XCL1	Lymphotactin; SCM-1α; ATAC	1q23	5.60	3	93	“Mini”-CC 1	XCR1
XCL2	XCL2	SCM-1β	1q24.2	3.23	3	93	“Mini”-CC 1	XCR1
**CX3C**								
CX3CL1	CX3CL1	Fractalkine; Neurotactin; ABCD-3	16q13	12.54	3	355^2^	“Mini”-CC 16	CX3CR1
**NOT ASSIGNED**								
MIF	MIF	Macrophage migration inhibitory factor, glycosylation-inhibiting factor	22q11.23	0.84	3	114		CXCR2, CXCR4; CXCR7, CD74

*Human gene and protein data were collected from the web sitesEntrezGene: http://www.ncbi.nlm.nih.gov/sites/entrez?db=gene and http://www.uniprot.org/, respectively*.

*^1^Only agonist receptors are indicated (adapted from Schall and Proudfoot, 2011)*.

*^2^Each chemokine domain of CXCL16 and CX3CL1 is constituted by 76 amino acids*.

Besides their structural classification, another organization of chemokines has been proposed based on their expression and their functional activity. This classification groups chemokines into three “families”: pro-inflammatory, homeostatic, and mixed function (Mantovani et al., 2006). Pro-inflammatory chemokines are up-regulated under inflammatory conditions and are involved in the leukocyte recruitment to inflamed sites. Homeostatic chemokines are expressed constitutively at non-inflamed sites and are involved in homeostatic migration and homing of cells in physiological conditions such as lymphocyte homing. Some chemokines have both properties, and are thus called mixed-function chemokines.

Chemokines act by binding specialized receptors on the target cell surface. These chemokine receptors are also grouped into four families, CXCR, CCR, XCR, and CX3R, based on the chemokine family they bind (Nomiyama et al., 2011). The entire group of chemokine receptors belongs to the seven transmembrane domain G-protein-coupled receptors that usually combine the receptor to the Gαi subunit of heterotrimeric G proteins. So far, around 25 human chemokine receptor genes have been identified (Table 2). Interestingly, 12 of these receptors are found on human chromosome 13 and stretched around 13.5 megabases. In addition, several decoy receptors have been reported to bind chemokine ligands without eliciting signal transduction. These comprise CXCR7, CCBP2, Duffy antigen receptor for chemokines (DARC), CCRL1, and CCRL2. The first chemokine receptor genes appeared in the most primitive vertebrate, agnathan lamprey (hagfish), around 480 million years ago (Nomiyama et al., 2011).

**Table 2:** **Human chemokine receptor genes**.

Name	Conventional name	Chromosome	Gene size (kb)	Number of exons	Number of amino acids	Ligands
**CXCR**						
CXCR1	IL8R1; IL8RA; CMKAR1	2q35	4.15	2	350	CXCL6, CXCL8,
CXCR2	IL8R2; IL8RB; CMKAR2	2q35	11.96	4	360	CXCL1, CXCL2, CXCL3, CXCL5, CXCL6, CXCL7, CXCL8, MIF
CXCR2P1	CXCR2P; IL8RBP	–		1		
CXCR3A	IP10-R; MigR; CMKAR3;	Xq13.1	2.60	2	368	CXCL4, CXCL4L1, CXCL9, CXCL10, CXCL11,
CXCR3B	IP10-R; MigR; CMKAR3;	–	–	2	415	
CXCR3-alt		–	–	2	267	
CXCR4	LAP3; LCR1	2q21	2.60	2	352, 356	CXCL12, MIF
CXCR5	BLR1; MDR15	11q23.3	12.43	2	372, 327	CXCL13
CXCR6	BONZO; CD186	3p21.26	4.87	2	342	CXCL16
**CCR**						
CCR1	CKR1; CMKBR1; MIP1aR	3p21	6.63	2	355	CCL3, CCL3L1, CCL3L3, CCL5, CCL7, CCL14, CCL15, CCL16; CCL23,
CCR2A	CMKAR2; CD182; CKR2B	3p21.31	7.18	2	374	CCL2, CCL7, CCL8, CCL13,
CCR2B	CKR2B	–	–	3	360	
CCR3	CKR3; CMKBR3	3p21.3	24.32	3	355	CCL2, CCL5, CCL7, CCL8, CCL11, CCL13, CCL15, CCL24, CCL26, CCL28,
CCR4	CKR4; CMKBR4; ChemR13	3p24	3.33	2	360	CCL17; CCL22
CCR5	CMKBR5; CKR5	3p21.23	6.06	3	352	CCL3, CCL3L1, CCL3L1, CCL4, CCL4L1, CCL4L2, CCL5, CCL7, CCL11, CCL13
CCR6	BN-1; DCR2; CKR-L3	6q27	27.33	3	374	CCL20
CCR7	BLR2; CMKBR7; EBI1	17q12-q21.2	11.71	3	378	CCL19, CCL21
CCR8	CKRL1; CMKBR8; CMKBTER1	3p22	3.97	2	355	CCL1
CCR9A	GPR-9-6; GPR28	3p21.3	16.67	4	359	CCL25
CCR9B	GPR-9-6; GPR28	–	–	–	357	CCL25
CCR10	GPR2	17q21.1-q21.3	2.42	2	362	CCL27, CCL28
**XCR**						
XCR1	CCXCR1; GPR5	3p21.3	7.68	2	333	XCL1, XCL2
**CX3CR**						
CX3CR1	CMKDR1; GPR13; CCRL1	3p21.3	18.24	4	355, 387, 362	CX3CL1
**DECOYRECEPTORS**						
CXCR7	RDC1; GPR159	2q37.3	12.61	2	362	CXCL11, CXCL12, MIF
CCRL1	CCR11; CCBP2; VSHK1; CCX-CKR; PPR1	3q22	5.29	2	350	CCL19, CCL21, CCL25
CCRL1P1	dJ509I19.4	6q23.3		1		
CCRL2	CKRX; CRAM-A; CRAM-B; HCR	3p21	2.29	3	344, 256	CCL5, CCL19
CCBP2	D6	3p21.3	57.81	3	384	CCL2, CCL3, CCL4, CCL4L1, CCL4L2, CCL5, CCL7, CCL11, CCL13; CCL17, CCL22,
DARC	Duffy; FY	1q21-q22	2.48	2	336, 338	CCL2, CCL5, CCL7, CCL11, CCL13, CCL15, CCL17, CCL18, CCL22, CXCL1, CXCL2, CXCL3, CXCL5, CXCL6, CXCL7, CXCL8, CXCL11
**FORMYL PEPTIDE RECEPTOR**						
FPR2	FPRL1; LXA4R; FMLP-R-II; FMLPX; FPR2A; FPRH1	19q13.3-q13.4	9.33	3	351	CCL23, Lipoxin A4, serum amyloid A, β amyloid peptide Aβ42, SAA, MMK, Hp-(2-20)

*Human gene and protein data were taken from the web sitesEntrezGene: http://www.ncbi.nlm.nih.gov/sites/entrez?db=gene and http://www.uniprot.org/, respectively*.

The chemokine/chemokine receptor system can be considered as a “puzzle” since many receptors have different chemokines as ligands and vice versa. However, thanks to the multiple combinations allowed, this system offers robustness. Indeed, even if one chemokine or receptor does not function, another one can replace it.

## Chemokines in Leukocyte Trafficking and Inflammatory Diseases

Leukocyte recruitment represents a fundamental episode during infection, in inflammatory disorders, such as atherosclerosis, as well as in autoimmune diseases, such as in psoriasis, rheumatoid arthritis, and chronic lung disease (Luster et al., 2005). Initially, leukocyte extravasation was described as a three-step process namely rolling, activation, and arrest. Recently, new insights have allowed defining a more complex process by adding several steps to the three original, including tethering (or capture), slow rolling, adhesion strengthening, spreading, intravascular crawling, and finally paracellular and transcellular transmigration.

Whereas capture and slow rolling are mediated by reversible and transient interactions between E-, L-, or P-selectin and ligands such as P-selectin glycoprotein-1 (PSGL-1), the adhesion of leukocytes to endothelial cells is mediated by the interaction of VCAM1 and ICAM-1, receptor for advanced glycation endproducts (RAGE), or mucosal vascular cell-adhesion molecule 1 (MADCAM1) with leukocyte integrins. The common structure of integrins is a non-covalently associated α and β subunit. So far, 16 α subunits and 8 β subunits have been identified, and various combinations form at least 22 heterodimers. The principal neutrophil β2-integrins are CD11a/CD18 (LFA-1) and CD11b/CD18 (Mac-1, CR3; Luo et al., 2007) although neutrophils can express p150,95/αxβ2 (CD11c/CD18) and at low level very late antigen (VLA) 4/α4β1 (CD49d/CD18; Zarbock et al., 2012). LFA-1 and Mac-1 have been shown to mediate neutrophil adhesion by interacting with ICAM-1 while αxβ2 is able to bind the N-terminal part of the alpha chain of fibrinogen (Loike et al., 1991; Diamond and Springer, 1993; Lum et al., 2002). During neutrophil adhesion, LFA-1 and Mac-1 appear to have sequential roles binding ICAM-1 under shear conditions (Neelamegham et al., 1998; Hentzen et al., 2000). In a two-step process neutrophils adhere first to ICAM-1 by interacting with LFA-1 and then Mac-1 acts as a stabilizer of the LFA-1/ICAM-1 bond.

The transition of rolling to leukocyte arrest and activation is triggered by chemokines such as CXCL1/GRO-α while others like CCL2/MCP-1 *per se* are rather promoting transmigration. Arrest of rolling leukocytes is triggered by an increase in the affinity of integrins by chemokines (Ley et al., 2007; Chavakis et al., 2009).

Different cell types, such as mesenchymal stem cells, endothelial cells, and circulating blood cells including leukocytes or platelets produce and release a broad range of chemokines and other chemoattractants that facilitate and enhance the recruitment of leukocytes. Some of these pro-inflammatory mediators circulate in the plasma, others are only found in the inflamed tissue, and yet others are presented on endothelial cells. Furthermore, additional to direct endothelial deposition from the luminal side, chemokines are transported via caveolae through the endothelium and presented to the apical side of the cell instead of diffusing through endothelial cell junctions (Pruenster et al., 2009). This transcytosis requires the DARC (Middleton et al., 1997). Recently, a new mechanism has been highlighted introducing the concept of lymphocyte transendothelial migration by intraendothelial vesicle-stored chemokines beneath the apical membrane (Shulman et al., 2011).

Chemokines bind chemokine receptors expressed on leukocytes to induce activation. In addition, most chemokines are also able to bind extracellular matrix components, including glycosaminoglycans (GAGs), to get immobilized and be presented to leukocytes. This is essential in order to avoid to be swept away under flow conditions from the cell surface. This coimmobilization with adhesion molecules will promote leukocyte activation, adhesion, and migration.

The following section will give several examples of chemokine contribution in leukocyte trafficking and in inflammatory diseases with a particular focus on vascular inflammatory diseases.

### Chemokines in platelets

As outlined above, activated platelets are able to release chemokines as well as a battery of different mediators to modulate inflammation. Thus, platelets have been found to be involved in different diseases with an inflammatory component such as obesity, acute lung injury, or coronary artery disease where they interact with both endothelial cells and leukocytes leading to a diversity of effects (van Gils et al., 2009; von Hundelshausen et al., 2009). Platelets release chemotactic cytokines stored in α-granules upon activation. *Inter alia*, CXCL4/PF4, CCL5/RANTES, CXCL7/NAP-2, CXCL12/SDF-1, CXCL1/GRO-α, or CXCL5/ENA-78 are able to mediate the endothelial adhesion of different cells including monocytes, neutrophils, and progenitor cells (Lievens and von Hundelshausen, 2011).

Platelets secrete CXCL4 which is the first and most abundant chemokine identified in releasates from activated platelets and which is involved in a wide range of physiological processes such as proliferation and angiogenesis. This chemokine is also involved in numerous pathological processes. High levels of CXCL4 are positively correlated with Crohn’s disease activity index (Simi et al., 1987). In Heparin-induced thrombocytopenia (HIT), autoantibodies developed against high molecular complexes of CXCL4/heparin or CXCL4/GAG side chains. The presence of HIT antibodies can lead to platelet activation and depletion through platelet consumption in venous thrombosis (Greinacher, 2009). CXCL4 exerts chemotactic activities on different cells including neutrophils, monocytes (Deuel et al., 1981), and activated T-lymphocytes in a pertussis toxin-sensitive manner (Mueller et al., 2008). Recently, CXCL4 has also been shown to be able to induce a specific macrophage type with specific phenotypic and functional characteristics (Gleissner, 2012). Moreover, it promotes adhesion of neutrophils on endothelial cells (Petersen et al., 1999). Although CXCL4 has been reported to bind to and stimulate CXCR3 and a splice variant thereof (CXCR3B), the functional importance of these two receptors for the biological activity of CXCL4 is not clear. For instance, a recent report found CXCL4 to be involved in ligand driven monocyte down-regulation of chemokine receptors CCR1, CCR2, and CCR5 by releasing the respective ligands (CCL2-4) from CXCL4-activated monocytes in absence of CXCR3 highlighting the connection between platelets and monocytes (Schwartzkopff et al., 2012). CXCL4 can induce exocytosis and firm neutrophil adhesion to endothelium when incubated with the appropriate co-stimuli. A contribution of CXCL4 in cardiovascular diseases has been described in both human and mouse where CXCL4 has been found in the endothelium, neovasculature, macrophages, and calcified regions of atherosclerotic carotid arteries (Pitsilos et al., 2003). Moreover, a strong positive correlation between both luminal and neovascular CXCL4 staining and coronary artery disease and between CXCL4 in macrophages and the presence of symptomatic atherosclerotic disease has been found. In a murine model of atherosclerosis, the knock-out of CXCL4 has been shown to exert an atheroprotective effect reducing atherosclerotic lesion formation (Sachais et al., 2007). Activation of platelets results in a release of stored-P-selectin -CXCL4 and CCL5 from granules. CCL5 has been detected on the luminal surface of atherosclerotic murine and human carotid arteries or neointimal lesions after arterial injury and can be deposited on inflamed or atherosclerotic endothelium by activated platelets, thereby triggering monocyte recruitment under flow (von Hundelshausen et al., 2001; Schober et al., 2002). The deposition of platelet chemokines can be facilitated by platelet-derived microparticles (Mause et al., 2005). Injection of activated platelets into the tail vein of atherosclerosis prone mice results in exacerbated atherosclerotic lesions and increased endothelial deposition of CXCL4 and CCL5 dependent on the presence of P-selectin (Huo et al., 2003). Therefore, platelet adhesion molecules such as P-selectin are mediating transient interactions with endothelial cells enabling a local delivery of soluble chemokines. We have discovered that heterophilic interactions between CXCL4 and CCL5 (see below) are responsible for enhanced monocyte recruitment into the arterial wall which explains to a certain extent why activated platelets are strong promoters of atherosclerosis. Peptides inhibiting the association of CXCL4 and CCL5 decrease atherosclerosis and macrophage content of lesions (von Hundelshausen et al., 2005; Koenen et al., 2009).

A non-allelic variant form of CXCL4, called CXCL4L1 or PF4ALT which differs only in three amino acids in the C-terminal α-helix of the protein has been identified in different kind of cells including leukocytes, endothelial, or smooth muscle cells (Lasagni et al., 2007). CXCL4L1 is capable of inducing endothelial cell chemokinesis and has been characterized as a potent anti-angiogenic regulator similar to CXCL4. Important differences of CXCL4L1 and CXCL4 are a lower affinity for CCL5 (Sarabi et al., 2011) and GAGs, e.g., heparin (Dubrac et al., 2010). Substantiating the role of CCL5–CXCL4 heterodimers CXCL4L1 failed to increase CCL5-triggered monocyte adhesion (Sarabi et al., 2011). The decreased affinity of CXCL4L1 compared to CXCL4 may have critical implications for cell adhesion since CXCL4L1 will not be retained at its site of expression. The existence of heparan sulfate in the subendothelial extracellular matrix has been found to regulate the arrest function of CCL5 and CCL4/MIP-1β (Gilat et al., 1994). On the endothelial surface under flow conditions, both platelet-derived and recombinant CCL5 are able to bind to activated endothelium and to trigger the firm arrest and transmigration of monocytes (von Hundelshausen et al., 2001).

Moreover, the oligomerization of CCL5 is crucial for CCR1-mediated leukocyte arrest on inflamed endothelium but not for their transmigration via CCR5 (Baltus et al., 2003). In bronchial mucosa of patients with chronic obstructive pulmonary disease (COPD), CCL5, and to a lesser extent CXCL7, have been found to be the most abundant chemokine expressed in the bronchial epithelium and are associated with an increase of neutrophil activation (Di Stefano et al., 2009).

CXCL12 or SDF-1α, which is the ligand for CXCR4 and CXCR7, has both proatherogenic and antiatherosclerotic properties (Weber et al., 2011). Blocking the CXCR4–CXCL12 axis leads to a release of different leukocyte subsets into the circulation. In this context, monocytosis and neutrophilia are conditions positively correlated with the development and severity of atherosclerosis. On the other hand has CXCL12 been demonstrated to be crucial for the healing of arterial lesions by the regenerative capacity of progenitor cells which are attracted to adhere be CXCL12 (Massberg et al., 2006). In addition to its production by platelets, CXCL12 is expressed in the bone marrow and in cells directly relevant to atherogenesis, including endothelial cells, smooth muscle cells, and leukocytes, which enables it to regulate the trafficking and localization of immature and maturing leukocytes, including bone marrow stem cells, neutrophils, T cells, and monocytic cells (Abi-Younes et al., 2000; Zeiffer et al., 2004; Stellos et al., 2009). Furthermore, CXCL12 has been thought to play a pro-inflammatory role in various autoimmune diseases, especially in rheumatoid arthritis and nephritis, in murine lupus erythematosus as well as in ongoing experimental autoimmune encephalomyelitis (Meiron et al., 2008; Karin, 2010). Recently, changes in CXCL12 signaling patterns have been found to be necessary for bone marrow neutrophil mobilization and are involved in polymicrobial sepsis, where its inhibition resulted in peritoneal cavity neutropenia (Delano et al., 2011). While both CCL5 and CCL7/MCP-3 are able to activate and to induce the chemotaxis of eosinophil and basophil granulocytes in allergy (Baggiolini and Dahinden, 1994), CCL11/Eotaxin has been found to be a powerful attractant for eosinophils and has also been identified in atherosclerotic lesions (Baggiolini et al., 1997; Haley et al., 2000).

The expression of CXCL7 is restricted to the platelet-lineage. Proteolytic cleavage of the carboxy-terminal part of pro-platelet basic protein (PPBP) and the proteolytic removal of the N-terminal part of PPBP produces two other chemokines namely connective tissue-activating peptide III (CTAP-III) and beta-thromboglobulin (beta-TG; Walz and Baggiolini, 1990; von Hundelshausen et al., 2007). Dependent on CXCR2, CTAP-III, and CXCL7 promote neutrophil and monocyte adhesion to human endothelial cells under flow conditions, respectively (Schenk et al., 2002; Baltus et al., 2005). The chemotactic potential of CXCL7 is also enhanced in COPD patients (Traves et al., 2004).

CXCL5/ENA-78 has been shown to act as a potent chemoattractant and activator of neutrophil function via CXCR2 (Ahuja and Murphy, 1996). CXCL5 has also been found to be strongly correlated with the number of neutrophils in patients with acute respiratory distress syndrome (Goodman et al., 1996).

During early atherosclerosis, CXCL1/GRO-α immobilized on the surface of endothelial cells via heparin proteoglycans induces the firm adhesion of rolling monocytes expressing CXCR2 (Schwartz et al., 1994; Huo et al., 2001; Boisvert et al., 2006). Moreover, a recent study has shown that *in vivo*, lysophosphatidic acid increased the progression of atherosclerosis and recruited leukocytes to the vessel wall during early atherogenesis via lysophosphatidic acid receptor-mediated release of endothelial CXCL1 (Zhou et al., 2011). A study conducted on elderly COPD patients has also indicated that CXCL1 might be a relevant candidate biomarker for this disease (Tsai et al., 2010).

### Chemokines in mast cells

In addition to their role as sentinels in the recognition of pathogens, mast cells (like platelets) are able to communicate with immune cells facilitating the recruitment of leukocytes to sites of infection. Indeed, mast cells are able to produce different chemokines including CCL4, CXCL8, or CCL11 assisting in the recruitment of CD8^+^ T cells, eosinophils, and natural killer cells, respectively (Abraham and St John, 2010).

CCL3/MIP-1α and CCL4/MIP-1β can initiate diverse cellular responses that regulate both acute and chronic inflammation via their interaction with CCR1 and CCR5. In addition, proteoglycan-bound CCL4 is used to effectively activate and induce the adhesion of circulating lymphocytes for their extravasation through lymph node endothelium (Tanaka et al., 1993). The quaternary structures of CCL3 and CCL4 are decisive for their biological activity. Aggregation of CCL3 and CCL4 can be considered as polymerization processes of MIP-1 dimers, which constitute the basic unit of MIP-1 proteins. MIP-1 monomers form dimers of the CC-type by creating an anti-parallel β-sheet of the N-termini (Lodi et al., 1994; Czaplewski et al., 1999; Ren et al., 2010). MIP-1 dimers associate to polymers consisting up to 50 units forming a double helixed rod like structure. Polymerization of MIP-1 protects MIP-1 from proteolytic degradation while the positively charged region of MIP-1, which is crucial for the receptor binding, is buried. The continuous and slow release of monomers from the polymer leads to a shallow gradient with a long gradient and effective range for leukocyte recruitment.

CXCL8/interleukin-8/IL8 has been found in intracellular granules from skin mast cells and mast cell lines (Möller et al., 1993). Recently, Kim et al. (2010) have shown that CXCL8 synthesis is induced via the leukotriene B4/leukotriene B4 Receptor 2 pathway in response to IL-1β in human primary mast cells and mast cell line HMC-1. CXCL8 released by mast cells is implicated in the selective chemotaxis of CXCR1-expressing natural killer cells (Burke et al., 2008). CXCL8 also induces neutrophil migration and activation by binding to G-protein-coupled receptors on their surface, namely human CXCR1 and CXCR2 (Wuyts et al., 1998). During inflammation, CXCL8 is produced and presented to the endothelial surface in association with GAGs. In a recent study, using obligate monomeric and dimeric IL8 mutants, the oligomerization state of CXCL8 was shown to have an influence on the kinetics of the neutrophil extravasation. The dimeric form initiated a fast robust but short lived vascular efflux whereas the monomeric form resulted in a weaker but longer-lasting response (Das et al., 2010). Also, this chemokine is among the most important in the recruitment of inflammatory cells, mostly neutrophils, in COPD (Barnes, 2004).

### Chemokines in dendritic cells: CCL17 as example

CCL17/TARC (thymus and activation regulated chemokine) together with CCL22/MDC (macrophage-derived chemokine) are expressed in relevant amounts by mature dendritic cells but occur as well in other cell types such as fibroblasts. CCL17 is constitutively expressed in the thymus (Saeki and Tamaki, 2006). CCL17 is a ligand for CCR4, which is predominantly expressed on Th2 lymphocytes, basophils, and natural killer cells. Recently, dendritic cell-derived CCL17 has been found to be critical in atherosclerosis (Weber et al., 2011). Indeed, deficiency of CCL17 in Apoe^−/−^ mice results in a reduction of the plaque formation in aortic root since CCL17 inhibits the expansion of atheroprotective Tregs and attracts CD4^+^ and CD3^+^ T cells.

### Membrane-bound chemokines

In addition to different types of cells such as T cells, macrophages, cytokine-induced smooth muscle cells, and endothelial cells, CXCL16/SR-PSOX has been recently identified for the first time in platelets (Seizer et al., 2011). This protein constitutes an atypical chemokine because it is expressed as a cell surface bound molecule but is also found in a soluble form after shedding. CXCL16 has also been involved in different diseases. Thus, a low plasma concentration of CXCL16 has been associated with coronary artery disease and has been found in atherosclerotic lesions in human and mice (Wuttge et al., 2004; Sheikine and Hansson, 2006). *In vivo* and *in vitro*, homocysteine, a homolog of cysteine that can promote atherosclerosis (Harker et al., 1976), has been found to stimulate CXCL16 production and deposition on the surface of endothelial cells via both production of ROS and a PPARγ-dependant pathway, thereby increasing adhesion of lymphocytes to endothelial cells (Postea et al., 2008).

Like CXCL16, CX3CL1 is an atypical multimodular chemokine that exists both in a membrane-tethered or soluble form. The immobilized form consists of a chemokine domain anchored to the plasma membrane through an extended mucin-like stalk, a transmembrane helix, and an intracellular domain. Besides, CX3CL1 has an anti-apoptotic and a proliferative effect on smooth muscle cells (White et al., 2010). Previous data have shown that CX3CL1 could serve as an adhesion molecule (Fong et al., 1998; Goda et al., 2000). However, more recent data indicated that, although CX3CL1 might mediate leukocyte adhesion, this phenomenon occurred only under low shear force and not under physiological conditions (Kerfoot et al., 2003). Regarding endothelial cells, CX3CL1 is expressed on the surface of IFN-γ/TNF-α-activated HUVEC and promotes leukocyte adhesion to atherosclerotic mouse arteries *in vivo* and under arterial flow *in vitro*. More precisely, CX3CL1 expressed by inflamed endothelial cells is recognized by CX3CR1 on activated platelets. Ligation of platelet CX3CR1 results in platelet activation and subsequent exposure of P-selectin on the surface of adherent platelets (Schulz et al., 2007). The inflamed CX3CL1-expressing endothelial cells can also recruit the non-classical subset of monocytes which highly express CXC3CR1 (Geissmann et al., 2010). Under homeostatic conditions, the disruption of the CX3CL1–CX3CR1 axis leads to a specific reduction of circulating non-classical monocytes in mice (Landsman et al., 2009). Addition of full-length recombinant soluble CX3CL1 to human monocytes has also shown to decrease apoptosis triggered by serum deprivation or treatment with 7-β-hydroxycholesterol. This reduction of apoptosis occurred in both CX3CR1-expressing CD14^++^CD16^−^ and CD14^+^CD16^+^ monocyte subsets. However, the precise mechanism is still unclear. A recent study shows that platelets over-expressing CX3CR1 on their surface are recruited alone or in association with monocytes to the site of inflammation. This phenomenon might contribute to an acceleration of atherosclerotic lesions (Postea et al., 2012).

### Chemorepulsion

Another aspect to take into consideration in the involvement of chemokines in leukocyte trafficking is the fact that chemokines could favor a “flight” of leukocytes from a tissue to reach the blood circulation or another tissue. In this case, leukocytes might run away from a chemokine gradient. This reverse migration from a peak concentration of chemokine is named chemorepulsion or fugetaxis. However, chemorepulsion refers more to a mediator that, depending on its concentration, can either repel or recruit cells using the same receptor. This phenomenon has been comprehensively studied in the context of T-cell trafficking during the process of thymic emigration and for which an extensive review has been recently published (Bunting et al., 2011). It has been suggested that chemorepulsion could participate in the thymic egress of human thymocytes. Thus, high concentration of CXCL12 has been shown to repulse human single positive thymocytes *in vitro* and this “run away” could be abolished using a neutralizing CXCL12 antibody (Poznansky et al., 2000). Moreover, this chemokine has also been shown to be a chemorepulsive agent of firm adhesion to activated pancreatic islet microvascular endothelium for both diabetogenic CD4 and CD8 T cells from NOD/LtJ mice. This repulsion results in a decrease of T-cell integrin activation in a CXCR4-independent manner (Sharp et al., 2008). Using a modified flow chamber containing a transwell insert on which HUVECs are cultured, Lee et al. (2009) have shown that T cells that have extravasated in response to subendothelial CCL5 may intravasate after exposure to subendothelial CXCL12 under flow conditions. High concentration of a chemokine, as already observable in the typically bell shaped response upon increasing chemokine concentration, is an important factor. However the exact molecular mechanisms of chemokine-induced chemorepulsion are still ill defined. Using a CXCL12 model, Zlatopolskiy and Laurence (2001) postulated that chemokine-mediated repulsion would be triggered by an excess of free ligand in the vicinity of the cell that would lead to a dimerization of the receptor, followed by an internalization of the ligand/receptor complexes. Internalization, digestion of the ligand, and recycling of the receptors would be realized under the same way than during the chemoattraction process. The difference would take place through the localization of the recycled receptors. The reappearance of the internalized receptors may occur not on the apical side of the cell but on the basal side resulting in a reverse movement. Summarizing, the gradient dependent direction of a chemokine triggered movement is concentration dependent. Thus, at least two different signaling pathways have to exist at the beginning, converging later again to reorganize the cytoskeleton for cell polarization and movement. Possible explanations for the chemorepulsion at high concentrations and chemoattraction at low concentrations are the chemokine dimerization at high concentrations, high- and low-affinity binding sites for chemokines on their cognate receptor, rapid recycling of GPCRs, apical rearrangements of recycled GPCRs, the oligomerization or homodimerization of GPCR with receptor and non-receptor proteins, and allosteric mechanisms.

## Genetic Variations in Chemokine Genes

Different studies have been carried out in order to evaluate the relationship between chemokine/chemokine receptor genes and inflammatory diseases including cardiovascular diseases. Table 3 provides several examples illustrating the association of chemokine/chemokine receptor polymorphisms with cardiovascular diseases.

**Table 3:** **Examples of chemokine/chemokine receptor single nucleotide polymorphisms (SNP) associated with cardiovascular diseases**.

Gene	SNP	Associated with	*p*-Value	References
*CCL2*	rs1024611	Myocardial infarction	0.005 and 0.009^a^	McDermott et al. (2005)
			<0.001 and 0.001^b^	
		Coronary artery disease	<0.005	Szalai et al. (2001)
*CCL5*	rs2107538	Acute coronary syndrome	0.0073	Simeoni et al. (2004)
		Coronary artery disease	0.0038	
*CCL11*	rs1129844	Myocardial infarction	0.012 and 0.008^c^	Zee et al. (2004)
*CXCL5*	rs352046	Acute coronary syndrome	0.005	Zineh et al. (2008)
*CXCL8*	rs4073	Acute coronary syndrome	0.004	Zhang et al. (2011)
*CXCL12*	rs1746048	Myocardial infarction (early onset)	1 × 10^−8^	Kathiresan et al. (2009)
		Atherosclerosis severity and progression	0.009	Kiechl et al. (2010)
		Coronary artery disease	3 × 10^−10^	Schunkert et al. (2011)
	rs1801157	Myocardial infarction	0.007	Luan et al. (2010)
	rs501120	Coronary heart disease	1.4 × 10^−6^	Franceschini et al. (2011)
		Myocardial infarction	0.002	Qi et al. (2011)
		Coronary artery disease	9.46 × 10^-8^	Samani et al. (2007)
*CCR2*	rs1799864	Heart failure	0.015	Ortlepp et al. (2003)
		Myocardial infarction	0.007	Ortlepp et al. (2003)
			0.054^d^	Petrkova et al. (2003)
	rs34948438	Myocardial infarction	0.0013^e^	Karaali et al. (2010)
			0.0016^f^	
*CCR5*	rs333	Myocardial infarction	0.001	Kallel et al. (2012)
			0.0013	Karaali et al. (2010)
		Severe calcific aortic stenosis	0.037^g^	Ortlepp et al. (2004)
		Myocardial infarction	0.003^h^	Singh et al. (2012)
*CX3CR1*	rs3732379	Coronary artery disease	0.03	McDermott et al. (2001)
		Acute coronary syndrome	0.001	Moatti et al. (2001)
		Single in-stent restenosis	0.006	Niessner et al. (2005)
		Recurrent in-stent restenosis	0.011	
		Myocardial infarction	0.006^i^	Singh et al. (2012)

*^a^In multivariable adjustment and multivariable adjustment of pooled-sex cohort, respectively*.

*^b^In multivariable adjustment and multivariable adjustment of male cohort, respectively*.

*^c^In an age and smoking and body mass index, hypertension, diabetes, and randomized treatment assignment adjusted recessive model of inherence, respectively*.

*^d^In female cohort*.

*^e^In patients carrying CCR5 rs34948438 wildtype (wt)/deletion (Δ) genotype*.

*^f^Individuals carrying the CCR5 rs34948438 heterozygote or homozygous variant genotype (Δ/Δ + wt/Δ)*.

*^g^In patients carrying the CCR5 rs333 SNP or CTGF -447C allele*.

*^h^In individuals carrying both CCR5 rs1799987 and rs333 SNPs*.

*^i^In individuals carrying both CX3CR1 rs3732378 and rs3732379SNPs*.

In order to identify genes involved in cardiovascular diseases and before the emergence of genome-wide association studies (GWAS), many efforts have been undertaken with gene candidate studies. In these studies several chemokine or chemokine receptor gene candidates have been found to be associated with cardiovascular diseases. For instance, a polymorphism in the promoter region of the *CCL5* gene called rs2107538 has been found associated with coronary artery disease (Simeoni et al., 2004). However, an extensive analysis based on the MONICA/KORA Augsburg Case-Cohort, Athero-Express, and CARDIoGRAM Studies has been recently carried out. Though an association between high CCL5 levels and an unstable plaque phenotype has been found, no associations of either CCL5 serum levels or its content in carotid plaques or its different genotypes with CAD or other coronary events has been established (Herder et al., 2011). The result of this study suggests that CCL5 protein levels and its gene variants might not be considered as biomarkers for the risk of coronary events in humans. As discussed by Altshuler et al. (2008), studies of candidate genes are performed on specific variants that have a small *a priori* probability of being disease-causing. Those studies are also able to generate false positives due to the lack of knowledge of the genetic background of cases and controls. This could explain the low reproducibility in candidate gene studies and lack of recovery between GWAS and candidate gene studies.

Amongst the different GWAS for cardiovascular disease performed during the last years, chemokine *CXCL12* gene polymorphisms have been associated with CAD (e.g., Samani et al., 2007; Kathiresan et al., 2009; Franceschini et al., 2011; Schunkert et al., 2011). In addition, the study conducted by Mehta et al. (2011) found the CAD risk locus 10q11 to regulate the level of CXCL12 transcripts.

## Chemokine Synergism by Heteromerization

The regulation of chemokine activity during initiation and development of inflammatory diseases is crucial to reach a fast and directed response. There is evidence that the activity of chemokines can be modulated by posttranslational processing (reviewed by Proost et al., 2003) and synergistic cytokines, e.g., IFN-γ (Mortier et al., 2011). Especially at the early phase of inflammation the concentration of a specific chemokine might not be high enough for a sufficient cell response. Hence synergism would aid to speed up the chemokine-induced response of leukocyte migration and to increase combinatorial specificity (Gouwy et al., 2005; Paoletti et al., 2005). A mixture of low concentrated individual synergizing chemokines behaves like the receptor agonist at an adequate concentration. Although the synergism of some single chemokines has been explored, so far a complete overview how many chemokines are involved is still elusive. It was previously shown that chemokine receptor induced chemotaxis may be enhanced by addition of chemokines which have *per se* no effect are not cognate ligands of the respective receptor and are called synergy-induced chemokines (Paoletti et al., 2005). Currently it is still unclear how this effect may be mediated in detail. Several underlying mechanisms are conceivable and can depend mainly on the respective chemokine partners and their receptors. It is possible that homeostatic and inflammatory chemokines that exhibit a different functional activity can form heteromers and act together in a synergistic way. Furthermore the signaling of GPCR-agonists can be enhanced by non-ligand CXC- and/or CC-type chemokines. Additionally, the GPCR-agonist mono and dimer equilibrium may regulate the signaling of the specific GPCR, which results in a different recruitment pattern of the target cells (Drury et al., 2011). It is of strong interest how the chemokine–chemokine interactions occur *in vivo* but it is difficult to find feasible approaches for a direct observation of the processes in living organisms. Some examples for a chemokine–chemokine synergism are given in the next parts.

### Chemokine heteromerization

Interaction between receptor agonist and non-ligand chemokines influences the activity of the chemokine receptor. All chemokines exhibit a typical tertiary structure homology which consists of a disordered N-terminus followed by three anti-parallel β-strands and the C-terminal α-helix. The quaternary structures of CC and CXC chemokines are different. Whereas the CXC-type forms dimers with a central β-sheet, the CC-type dimerizes through the interaction of both N-termini. In case of CC- and- CXC-type heteromers it is difficult to predict the proper structure. Our workgroup previously showed the synergistic interaction of CXCL4 and CCL5 to accelerate atherosclerosis by triggering monocyte arrest on endothelium (von Hundelshausen et al., 2005; Koenen et al., 2009). The synergistic effect is based on the heteromerization of these two chemokines, since peptides disrupting the heteromers abolish this synergism. Interestingly, the quaternary structure of the CCL5–CXCL4 complex features a CC-type heteromer, which exhibits paired N-termini, yet results in better receptor activation.

The response to CCR4 in skin-homing T-lymphocytes is enhanced by co-expressed chemokines in the inflamed skin. For example the CXCR3-agonist CXCL10 enhances the chemotaxis of CCR4-transfected preB-cells and T cells due to interaction with the CCR4-agonist CCL22 (Sebastiani et al., 2005). Further enhancement of the CCR4 activity evolves from the direct interaction of CCL22 with the CCR7-agonist CCL19. In addition, CCL22 was also shown to interact with the CCR3-agonist CCL7. In this last case, it was shown that a sequence of five amino acids of the first β-strand from CCL7, which contains two positively charged arginine residues, is needed to synergize with CCL22 and hence increases the CCR4 activation. In the same study a CCL4–CCL7 chimera lost the synergetic activity, being generated by substituting the first β-strand of CCL7 with that of the non-synergizing CCL4, lacking the positively charged amino acids. Thus the first β-strand of a chemokine, containing positive and hydrophilic amino acids, seems to have a crucial role in synergism and heteromer formation.

Furthermore monocyte recruitment is enhanced by the homeostatic chemokines CCL19 and CCL21 which are both CCR7-agonists. They synergize with CCL7 and CCL2 that result in an augmented CCR2 response to recruit monocytes (Kuscher et al., 2009). Interestingly the induced monocyte recruitment by CCL7 is enhanced 100 times by CCL19 and CCL21 whereas CCL2 showed less synergistic activity. By comparing a specific motif, comprising five amino acids in the first β-strand of all four chemokines, it has further been shown that CCL7 and CCL21 exhibit more positively charged amino acids which correlates with a higher synergistic effect confirming the importance of the first β-strand. Synergism of chemokines by heteromerization was also shown for other chemokines (Paoletti et al., 2005; Allen et al., 2007; Koenen et al., 2009). The authors suggest that heteromers of synergistically acting chemokines lead to a high affinity conformation of the respective receptor. Another study (Venetz et al., 2010) showed that heteromerization of CXCL12 with the inflammatory CXCR3-agonist CXCL9 results in a higher response of CXCR4-expressing T cells and malignant B cells on tumor vasculature.

### Antagonism by chemokine dimers

Even if the neutrophil migration toward CXCL8 is enhanced by different CXC- and also CC-chemokines, i.e., CCL2 and CXCL12, the dimerization of CXCL8 decreases its binding to CXCR1 (Fernando et al., 2004; Weber and Koenen, 2006). This effect might not be due to structural change but rather to a loss of conformational flexibility which leads to a low-affinity configuration. Thus the dimer is not competent enough to bind the receptor N-domain. Moreover, heteromerization of CXCL8 with CXCL4 reduces the chemotactic propensity of CXCL8 (Dudek et al., 2003). These heterodimers enhance the anti-proliferative effect of CXCL4 on endothelial cells in culture, and the CXCL8-induced migration of CXCR2 transfected Baf3 cells as well (Nesmelova et al., 2005; Weber and Koenen, 2006). Inhibition of CXCL8-induced monocyte arrest is evoked by CXCL4. This effect might also be due to a less flexible CXCL8 molecule that has a lower affinity for its receptor. However, the availability of the monomer-dimer equilibrium of CXCL8 is crucial to regulate tissue-specific neutrophil recruitment given that the recruitment profile differs due to altered GAG-binding interaction (Gangavarapu et al., 2012).

Recently, it could be shown that a monomeric or dimeric state of CXCL12 plays a crucial role for the CXCR4 activation and its mode of signaling (Ray et al., 2012). The dimeric CXCL12 activates recruitment of β-arrestin 2 to CXCR4 and chemotaxis of CXCR4-expressing breast cancer cells, whereas the monomeric CXCL12 promotes the CXCR4 signaling through Gαi and Akt. Furthermore, another recent study (Drury et al., 2011) demonstrated that monomeric CXCL12 compared with the dimeric variant exhibits more contact sites for CXCR4 and thus results in different receptor signaling. To our knowledge it has not been tested, but supposedly a different or even inverse activity, e.g., the chemorepellent activity of higher concentrated CXCL12, may well be dependent on the preponderance of CXCL12 dimers.

Not only chemokine heteromerization may influence receptor driven signal transduction but as well the homooligomeric state changes the biological activity by either buried receptor binding sites, e.g., in polymeric MIP-1 or the different kinetics of monomeric versus dimeric CXCL8. These insights will be helpful to develop specific drugs interfering with oligomerization motifs thereby suppressing or enhancing desired chemokine effects.

### Binding to GPCRs

In order that chemokine–chemokine partners unfold synergism it is suggested that first chemokine heteromers form and subsequently receptor binding follows. Besides the formation of a heteromeric chemokine complex, the binding to a receptor is required to mediate the synergistic effects. GAGs, as co-receptors of GPCRs, can also induce heteromerization of chemokines (Crown et al., 2006). In addition, it is speculated that instead of heteromerization, as mentioned above, different receptor binding sites for CCL2 and CCL7 are responsible for the synergistic activity as it was previously shown for CXCR3-agonists (Colvin et al., 2004).

Homeostatic chemokines like CCL21, CCL19, CXCL12, and CXCL13 are synergizing to promote a regulated lymphocyte trafficking across the lumen or basal lamina of high endothelial venules (HEVs) in lymph nodes. For example, CXCL12 augments through its receptor CXCR4 the CCR7-induced chemotaxis of T cells and therefore helps to transfer them across the HEVs without direct interactions with the CCR7-ligands CCL19 and CCL21 (Bai et al., 2009). Here the signaling through CXCR4 has a major impact because in T cells, deficient in CXCR4, no cooperative effect was observed. The synergistic effect is merely evident at suboptimal concentrations of the CCR7-ligands CCL19 and CCL21. In summary, CXCL12–CXCR4 signaling has the ability to cause a maximal T-cell response by a suboptimal CCR7-ligand concentration. A similar observation was also previously shown for CXCL13 (Paoletti et al., 2005; Bai et al., 2009). However, it is remarkable that the heteromerization of CXCL13 with CCR7-ligands is thought to be the responsible mechanistic reason for synergism, whereas synergy of CXCL12 with CCR7-ligands is independent of direct chemokine–chemokine interaction. In fact, it is assumed that CXCL12 increases the CCR7-signaling by ERK phosphorylation and actin polymerization in T cells (Bai et al., 2009). A similar conclusion was provided by van Damme’s group who showed that the synergism of CXCL8 or CXCL12 with CCL2 is mediated through CXCR1/2 (CXCL8) and CXCR4 (CXCL12; Gouwy et al., 2008, 2009). When the concentration of CCL2 is low CXCL8 helps to chemoattract monocytes. This requires binding of CXCL8 to CXCR1 and CXCR2. A further example is the synergism of CXCL12 with CCL2 where correct binding and signaling to CXCR4 and CCR2 is essential for synergistic interactions. Additionally a recent study has shown that CCR1-agonists like CCL5 and CCL3 are enhancing CXCR4-induced ERK phosphorylation and chemotaxis of mononuclear cells and it was further observed that this cooperative effect is inhibited by blocking CCR1 with specific antibodies and AMD3100 (Gouwy et al., 2011).

In summary, synergism of chemokines crucially depends on increased activation of the GPCR by heteromerization of ligand and non-ligand chemokines or cooperative interactions after chemokine activation of distinct GPCRs. Heteromerization of receptors has been observed. However the mechanistic role of these complexes in respect of ligand binding is still unclear, it might be that chemokine heteromers can stabilize and change the functional activity of receptor heteromers (Thelen et al., 2010; Kramp et al., 2011).

## Therapeutics

GPCRs as therapeutic targets have been reviewed extensively (Rek et al., 2009; Koenen and Weber, 2010b, 2011; Bennett et al., 2011; Schall and Proudfoot, 2011; Kanzler et al., 2012). A lot has already been done and it is still in progress to find appropriate therapeutic drugs, particularly for the prevention and treatment of HIV. Since chemokines and the subsequent receptor signaling are involved in many diseases, there is hope that good antagonists will increase the means to treat them.

The different interactions between chemokines, which result in a changed biological activity, can be used to find new targets against inflammatory diseases. There are several possibilities for therapeutically targeting chemokines involved in inflammation. In the next part, several examples are illustrating how to alter inflammatory properties by blocking heterophilic interactions, multiple chemokine axes, direct blocking of chemokine receptors as well as blocking of GAG-binding sites.

### Modified chemokines

Modifying the target chemokine is one option to create antagonists since changing the molecular structure leads to a different binding pattern and receptor response. Especially the N-terminal part is crucial for receptor signaling and thus a change in this domain can lead to alteration or loss of receptor activation. Variants of chemokines with an extended or modified N-terminal part are for example N-methylated CCL5 (Met-RANTES) or amino-oxypentane-RANTES which block the CCL5 receptors CCR1, CCR3, and CCR5 (Proudfoot et al., 1996; Elsner et al. 1997; Proudfoot et al. 1999; Veillard et al., 2004). In liver fibrosis and atherosclerosis it was shown that inhibiting CCL5 receptors through Met-RANTES was sufficient to reduce inflammation in mice (Veillard et al., 2004; Berres et al., 2010). This phenomenon was observed *in vivo* and *in vitro*. Another example is R6H-CXCL8, a variant of CXCL8 with substitutions on the conserved ELR-triad and CXC-motif which exclusively activates CXCR1 without effecting CXCR2. This is based on a distinct CXCL8 binding mechanism: the CXCR2 activation is mediated by the N-terminal ELR- and CXC-motif whereas the N-loop of CXCL8 is essential for CXCR1 activation (Sarmiento et al., 2011). Furthermore, the above mentioned CXCL8 variant displays anti-inflammatory properties since it activates CXCR1 by desensitization of the CXCR2 response in human neutrophils. In fact, this agonist could help to clarify the biological and physiological function, especially of CXCR1, in inflammatory diseases. Thus R6H-CXCL8 is a potential candidate as a therapeutic molecule.

### Glycosaminoglycan binding affinity

Most chemokines have the ability to bind GAGs located on the cell surface. The enhancement or reduction of this property can diminish the GPCR signaling by an indirect blockade of chemokine binding to its receptor, since GAGs are co-factors for GPCR activation. It is assumed that chemokines first bind the GAG co-receptor followed by GPCR activation.

A variety of chemokines was previously designed with altered GAG-binding affinities resulting in a loss of GPCR activation (Proudfoot et al., 2008; Shahrara et al., 2008; Rek et al., 2009). The activity of the pro-inflammatory chemokine CCL5 depends on the binding to GAGs. The substitution of positively charged residues into alanine in the 40s loop ([44AANA47]-CCL5mutant) results in defective heparin binding and loss of the ability to recruit monocytes. The heteromerization of both CCL5 variants leads to non-functional heteromers with a lack of GAG-binding efficiency (Johnson et al., 2004; Koenen and Weber, 2010b). Another study (Braunersreuther et al., 2008) additionally confirms [44AANA47]-CCL5 as a potential therapeutic agent against atherosclerosis. But in contrast to Met-RANTES, [44AANA47]-CCL5 does not directly abolish GPCR activation. Thus variations of CCL5 mutants acting in different ways can lead to anti-inflammatory properties by a direct blockade of the GPCR or by indirect inhibition through prevention of chemokine binding to GAGs on the cell surface. Another way to block chemokine activity using the affinity for GAGs is to design dominant-negative mutants with a higher GAG-binding affinity compared to the wild type chemokine (Brandner et al., 2009). H23K-RANTES showed attenuation of autoimmune uveitis in rats based on displacement of wild type CCL5 from its proteoglycan-co-receptor. Mutants with increased GAG-binding potential were designed for CCL2, as well. The PA508 mutant of CCL2 exhibits no ability for CCR2 activation but a fourfold higher GAG affinity compared to the wild type CCL2 (Piccinini et al., 2010). In a recent study in mice, PA508-CCL2 showed prevention of neointima formation and reduction of tissue damage after myocardial infarction without notable side effects. Therefore, it could be a candidate as a therapeutic agent in reducing restenosis in stents (Liehn et al., 2010). Additionally, a mutant of CXCL12 with a deficiency in heparan sulfate binding can still transduce signals through CXCR4 but is not able to promote transendothelial migration *in vitro*. *In vivo* experiments could further show that this mutant efficiently down-regulates the CXCR4 expression and desensitizes the chemotactic response toward CXCL12. Hence, this modified chemokine might work in anti-inflammatory therapies (O’Boyle et al., 2009).

### Small molecules and antibodies

The development of small molecules blocking GPCR activation is a powerful tool for the treatment of inflammatory diseases. Major efforts have been done to find drugs for blocking HIV infection. Maraviroc (Celsentri/Selzentry; Pfizer) was established as a functional anti-HIV drug by blocking CCR5 as important entry receptor. In inflammatory diseases, like atherosclerosis, TAK779 and nbI-74330 antagonists for CCR5 and CXCR9, respectively, represent suitable therapeutic agents (Koenen and Weber, 2010b). Antagonizing CXCR4 by TAK779 additionally blocks leukocyte trafficking induced by CXCL12 (Sohy et al., 2009). Recently, DF 2156A was introduced as a novel dual inhibitor of CXCL8 receptors CXCR1 and CXCR2 (Bertini et al., 2012). This dual function is based on a non-competitive inhibition resulting in a stabilized binding between DF 2156A and the two CXCL8 receptors due to formation of specific ionic bonds in the allosteric binding site (Bertini et al., 2012). Some CXCR2- and CCR2-specific antagonists (i.e., reparixin and MLN1202) have already been tested as therapeutic drugs in clinical trials, like MLN1202, which is a CCR2-blocking monoclonal antibody shown to reduce high-sensitivity CRP as surrogate parameter for atherosclerosis (Allegretti et al., 2008; Gilbert et al., 2011).

### Chemokine heteromerization

As mentioned before some chemokines inherently exhibit synergistic function toward other chemokines which mostly depends on heteromerization. Disruption or changing these critical heterophilic interactions might entail a decrease in the physiological response which has an impact on the degree of inflammation. A prominent example is the heterophilic interaction between CXCL4 and CCL5 which results in a synergistic enhancement of CCL5 induced signaling accompanied by increased monocyte recruitment to the inflamed endothelium (Koenen et al., 2009; Koenen and Weber, 2010a). Interruption of the chemokine heteromerization by cyclic peptides was shown to eliminate synergistic effect *in vitro* and *in vivo*. Recently it was shown (Grommes et al., 2012)that small peptide antagonists, disrupting CXCL4–CCL5 heteromer formation in mouse models of acute lung injury, result in improved lung edema, less neutrophil infiltration, and reduced tissue damage. Thus targeting heterophilic chemokine interactions can act as therapeutic approach by attenuating inflammatory disease in a mild way.

## Conclusion

It is still elusive which consequences the blockade of one chemokine has when entering clinical trials. For example, the dendritic cell-derived CCL17 could be identified as a catalyzer for atherosclerosis due to interference of Treg homeostasis in mice (Weber et al., 2011). Blocking CCL17 with an antibody abolished this pro-inflammatory effect. Nevertheless the blocking mechanism is unclear and which consequences the blocking has for other physiological signal cascades. For example the CXCL12–CXCR4 axis is crucial for the CXCL12-dependent recruitment of progenitor cells. Consequently a reduction of the CXCR4 level diminishes this important process in regeneration but inversely a decreased expression of CXCR4 was efficient to limit myocardial infarct size in mice (Liehn et al., 2011). Detailed knowledge and clarity of how a specific chemokine oligomerizes, binds to GAG and its GPCR as well as its interaction with other chemokines, with regard of the resulting signal cascade and immune response, are required.

Targeting GAG-binding sites of specific chemokines is a promising approach for developing drugs against chemokine driven diseases, given that the GPCR binding is not directly affected. Also the disruption of chemokine–chemokine interactions seems to become attractive, since synergistic effects can be prevented without reducing the function of the respective chemokine *per se*.

## Conflict of Interest Statement

The authors declare that the research was conducted in the absence of any commercial or financial relationships that could be construed as a potential conflict of interest.
